# Prevalence of *BRCA1* and *BRCA2* Germline Mutations in Patients of African Descent with Early-Onset and Familial Colombian Breast Cancer

**DOI:** 10.1093/oncolo/oyab026

**Published:** 2022-02-15

**Authors:** Elizabeth Vargas, Robert de Deugd, Victoria E Villegas, Fabian Gil, Lina Mora, Luis Fernando Viaña, Ricardo Bruges, Alejandro Gonzalez, Juan Carlos Galvis, Ute Hamann, Diana Torres

**Affiliations:** 1 Institute of Human Genetics, Pontificia Universidad Javeriana, Bogota, Colombia; 2 Molecular Genetics of Breast Cancer, German Cancer Research Center (DKFZ), Heidelberg, Germany; 3 CENTOGENE GmbH, Rostock, Germany; 4 Centro de Investigaciones en Microbiología y Biotecnología-UR (CIMBIUR), Facultad de Ciencias Naturales, Universidad del Rosario, Bogota, Colombia; 5 Unit of Clinical Epidemiology and Biostatistics, Pontificia Universidad Javeriana, Bogota, Colombia; 6 Cancer League, Cartagena, Colombia; 7 Centro Javeriano de Oncología, Pontificia Universidad Javeriana, Bogota, Colombia

**Keywords:** *BRCA1/2*, breast cancer, germline mutation, Afro-Colombian

## Abstract

**Background:**

Pathogenic germline mutations in the *BRCA1* and *BRCA2* (*BRCA1/2*) genes contribute to hereditary breast/ovarian cancer (OC) in White/mestizo Colombian women. As there is virtually no genetic data on breast cancer (BC) in Colombians of African descent, we conducted a comprehensive *BRCA1/2* mutational analysis of 60 Afro-Colombian families affected by breast/OC.

**Materials and Methods:**

Mutation screening of the complete *BRCA1/2* genes for small-scale mutations and large genomic alterations was performed in these families using next-generation sequencing and multiplex ligation-dependent probe amplification analysis.

**Results:**

Four pathogenic germline mutations, including one novel mutation, were identified, comprising 3 in *BRCA1* and one in *BRCA2*. The prevalence of *BRCA1/2* mutations, including one *BRCA1* founder mutation (c.5123C>A) previously identified in this sample set, was 3.9% (2/51) in female BC-affected families and 33.3% (3/9) in those affected by both breast and OC. Haplotype analysis of 2 *BRCA2*_c.2701delC carriers (one Afro-Colombian and one previously identified White/mestizo Colombian patient with BC) suggested that the mutation arose in a common ancestor.

**Conclusion:**

Our data showed that 2/5 (40%) mutations (including the one previously identified in this sample set) are shared by White/mestizo Colombian and Afro-Colombian populations. This suggests that these 2 populations are closely related. Nevertheless, variations in the *BRCA1/2* mutational spectrum among Afro-Colombian subgroups from different regions of the country were observed, suggesting that specific genetic risk assessment strategies need to be developed.

## Teaser

Pathogenic germline mutations in the *BRCA1* and *BRCA2* (*BRCA1/2*) genes contribute to hereditary breast and ovarian cancer in white/mestizo Colombian women, but there is virtually no genetic data on breast cancer in Colombians of African descent. This article provides a comprehensive *BRCA1/2* mutational analysis of 60 Afro-Colombian families affected by breast or ovarian.

**Implications for Practice**

The differences in the frequency and spectrum of mutations in *BRCA1/2* genes show considerable variation among ethnic groups. However, only limited data on the contribution of *BRCA1/2* mutations to hereditary breast/ovarian cancer in the African population are currently available and there is no information on the Afro-Colombian population. Therefore, more research of the genetic factors contributing to breast and/or ovarian cancer in Colombian families of African descent is needed, which will enable the detection of mutations to guide prevention and therapy, considering that Colombia is the second most ethnically diverse country in the Americas, after Brazil.

## Introduction

Breast cancer (BC) is a major public health concern worldwide, with an estimate of more 2 million newly diagnosed cases and 684.996 deaths in 2020.^1^ In Colombia, BC is the most frequent cancer among women, with incidence and mortality age-standardized rates of 48.3 and 13.1 cases per 100 000 people per year, respectively.^1^

The Colombian population is ethnically diverse as a result of interracial relationships between indigenous peoples, Spanish colonizers, and enslaved Africans.^2^. Its 3 main ethnic groups are the mestizos (53%), White European Colombians (20%), and African Colombians (25%).^3^ The Afro-Colombian population concentrated on the northwest Caribbean coast and the Pacific coast is the second largest population of African descendants in Latin America.^4^ It consists of 3 ethnic categories: Raizal, Palenquero, and Mulatte.^5^ Raizal refers to people derived from admixture on the Caribbean islands, mostly between English, Dutch, and African slaves and slaves to other Caribbean islands.^6-8^ The Palenquero population refers to descendants of enslaved Africans who fled and established isolated and anti-colonial residences, forming the village of Palenque de San Basilio’s. Mulatte refers to first-generation offspring of African and European ancestry.^5^

Approximately 10% of BC cases are due to genetic factors and associated with a family history, which can be attributed to germline mutations in BC susceptibility genes, in particular *BRCA1* and *BRCA2* (*BRCA1/2*).^9,10^ The contribution of *BRCA1/2* germline mutations to early-onset and hereditary breast/ovarian cancer (OC) in the Colombian population was previously described by us in 2 studies conducted among a total of 121 White/mestizo breast/OC-affected families and unselected patients with BC. In these studies, we reported on the prevalence of pathogenic *BRCA1/2* germline mutation and the identification of 4 small-scale founder mutations and 1 large deletion, 2 in *BRCA1* (HGVS/BIC: c.3331_3334delCAAG/3450del4 and c.5123C>A/A1708E) and 3 in *BRCA2* (c.1763_1766delATAA/1991del4, c.2808_2811delACAA/-3034del4, and ex1-14del), which accounted for 89% and 44% of all *BRCA1* and *BRCA2* mutations, respectively.^11,12^ The overall prevalence of *BRCA1/2* mutations was 16% in families with multiple female cases of BC and 21% in families affected by both breast and OC. Furthermore, 2 other previous studies on unselected patients with breast cancer and OC showed mutation frequencies of 1.2% and 15%, respectively.^13,14^

Currently, limited information is available on the contribution of the *BRCA1/2* genes to hereditary BC in the Colombian population of African descent. In a single previous study conducted regarding this ethnic group, screening for the 4 small-scale Colombian *BRCA1/2* founder mutations led to the identification of one *BRCA1* mutation in 60 Afro-Colombian families affected by breast/OC.^15^ To define the spectrum of mutations and to estimate mutation frequencies, we have now comprehensively analyzed the complete *BRCA1/2* genes for small-scale mutations and large genomic rearrangements (LGRs) in the same sample set using next-generation sequencing (NGS) and multiplex ligation-dependent probe amplification (MLPA).

## Materials and Methods

### Study Population

All Afro-Colombian families affected by breast/OC who were investigated in this study have previously been described.^15^ In summary, a total of 60 families comprising 51 affected by BC and 9 affected by both breast and OC were recruited at the Cancer Leagues in Cartagena, Quibdó, and San Andres Island, Colombia, from March to December 2016. Of the 60 index patients, 52 were Afro-Colombians and 8 were Raizales. All patients had previously been screened for the 4 Colombian *BRCA1/2* founder mutations (*BRCA1*/c.3331_3334delCAAG, *BRCA1*_c.5123C>A, *BRCA2/*c.1763_1766delATAA, and *BRCA2*/c.2808_2811delACAA) and a mutation carrier (*BRCA1*_c.5123C>A) was identified.^15^ All patients were selected for genetic *BRCA1/2* testing using NGS. They were classified into 3 categories based on the family history of cancer: group A1: families with one female case of BC diagnosed at or before 35 years of age; group A2: families with 2 or more BC cases diagnosed at any age; and group A3: families with one or more female BC cases and one or more OC cases diagnosed at any age.

Information on ethnicity, as well as personal and family history of BC, was obtained from all study participants through self-administered questionnaires. Written informed consent was provided by all study participants. The research protocol was approved by the Ethics Committee of the Pontificia Universidad Javeriana in Bogota, Colombia (approval number: 06/2019).

### DNA Isolation and *BRCA1/2* Mutation Analyses

Genomic DNA was extracted from 14 mL of peripheral blood collected into an ethylenediaminetetraacetic acid tube using the salting-out extraction method.^16^ The *BRCA1/2* genes were screened for small-scale mutations using NGS (CENTOGENE, Rostock, Germany) in the 60 index patients. Genomic DNA was enzymatically fragmented and regions of interest were selectively enriched using capture probes targeted against coding regions of the panel genes. Libraries were generated with Illumina-compatible adaptors and sequenced on an Illumina platform. For the *BRCA1/2* panel, the entire coding region of the *BRCA1/2* genes including 10 bp of flanking intronic sequences was targeted. The panel included analysis of all reported disease-causing deep intronic and regulatory mutations described outside the coding ±10 boundary. Owing to the limitations of this method, all the targeted sequences within the requested panel may not be covered. Missing regions or regions of poor quality were then further examined using Sanger sequencing to achieve 100% coverage. Clinically relevant variants identified by NGS were validated according to quality standards. Raw sequence data analysis including base calling, demultiplexing, alignment to the hg19 human reference genome (Genome Reference Consortium GRCh37), and variant calling was performed using validated in-house software. The American College of Medical Genetics and Genomics nomenclature guidelines were used to annotate identified variants. The Human Gene Mutation Database constituting a comprehensive collection of published germline mutations, and the ClinVar database and CentoMD were used to determine the biological significance of the identified variants.

Families negative for small-scale mutations were analyzed for LGRs using the MLPA technique, as previously described.^17^ In summary, MLPA analysis was performed using probe mix P002 and P087 for *BRCA1* (Catalogue No. P002-100R) and P045, P090, and P077 (Catalogue No. P090-100R) for *BRCA2*, in accordance with the manufacturer’s instructions (MRC Holland, Amsterdam, The Netherlands). The separation and relative quantification of the amplified product was achieved using the Beckman CEQ 8000XL DNA Analysis System (Beckman Coulter, Fullerton, CA). The variation in peak height was evaluated by comparing each sample with normal controls using SeqPilot software version 3.5.2 (JSI Medical Systems, Kippenheim, Germany).

### Haplotype Analysis

Two patients with BC carrying the *BRCA2*_c.2701delC mutation (one Afro-Colombian carrier identified in the present study and one previously identified White/mestizo carrier^11^) were scored for allele sharing indicative of a common ancestor. Haplotype analysis was performed at the 4 extragenic microsatellite loci D13S290, D13S260, D13S171, and D13S267, flanking the *BRCA2* gene.^18^ Microsatellite alleles were identified by automated fluorescent-based fragment detection from amplified PCR products using a CEQ 8000 XL DNA Analysis System (Beckman Coulter).

### Statistical Analysis

Comparison of the age of diagnosis between *BRCA1/2* carriers and noncarriers was performed using the Wilcoxon rank-sum test with continuity correction. Results were judged as statistically significant at a *P*-value of .05 or less. All statistical analyses were performed using R software (https://www.r-project.org/).

## Results

### Description of the Afro-Colombian Families

The present study included 60 index cases from 60 Afro-Colombian families affected by breast/OC. Three patients had been diagnosed with BC before 35 years of age (5%); 48 belonged to families with at least 2 BC cases (80%) and 9 to families with both breast and OC (15%). The median ages of disease onset differed between *BRCA1/2* carriers (*n* = 5, including the previously identified *BRCA1*_c.5123C>A carrier)^15^ and noncarriers (*n* = 55; 40 years, range 30-45 vs 51 years, range 33-70 years, *P* = .019 by Wilcoxon rank-sum test).

### *BRCA1/2* Mutations and Frequencies

Using NGS analysis, 4 deleterious mutations were identified: 3 in *BRCA1*, including one novel mutation, and one in *BRCA2*. Additionally, 3 distinct *BRCA2* variants of uncertain clinical significance were detected (Supplementary Table S1). The *BRCA1/2* mutation frequency including the known *BRCA1*_5123C>A mutation was 3.9% (2/51) in families with female BC cases and 33.3% (3/9) in families affected by both breast and OC. Descriptions of the *BRCA1/2* mutations and frequencies by risk group are presented in Tables 1 and 2.

**Table 1.** *BRCA1/2* mutation frequencies in the 60 Afro-Colombian families affected by breast/ovarian cancer by risk groups.

Risk group	Family phenotype	No. of families	No. (%) of families with mutations in		
			*BRCA1*	*BRCA2*	*BRCA1/2*
	Families with female BC cases	51	2 (3.9)	0 (0.0)	2 (3.9)
A1	1 case ≤35 years	3	0 (0.0)	0 (0.0)	0 (0.0)
A2	Multiple cases	48	2 (4.2)	0 (0.0)	2 (4.2)
A3	Families with both BC and OC	9	2 (22.2)	1 (11.1)	3 (33.3)
	≥1 BC and ≥1 OC, at any age				
	All families	60	4^a^ (6.7)	1 (1.7)	5 (8.3)

Including the *BRCA1*_c.5123C>A mutation previously identified.^15^

BC, breast cancer; OC, ovarian cancer.

**Table 2.** Description of the *BRCA1/2* mutations in Afro-Colombian families affected by breast/ovarian cancer.

Family	Ethnic group	Gene	Mutation nomenclature			No. of BIC entries^a^						No. of ClinVar Entries^g^	Total entries^h^
			BIC^a^: Genomic Level	HGVS^b^: genomic level	HGVS:protein level	Asian descent	European descent	African descent^c^	Hispanic descent^d^	Unknown^e^	Total^f^		
*Deleterious BRCA1/2 mutations*													
A004	Afro	*BRCA1*	IVS5-12A>G	c.213-12A>G	—	1	16	1^j^	2^l^	8	28	55	83
A012	Afro	*BRCA1*	4282insA	c.4163_4164insA	p.Ser1389Glufs	0	0	1^j^	0	0	1	4	5
A035	Afro	*BRCA1*	5242C>A^i^	c.5123C>A	p.Ala1708Glu	0	15	1^j^	12	19	47	77	124
A036	Raizal	*BRCA1*	—	**c.4203delT**	**p.Gln1401Hisfs**	0	0	1^k^	0	0	1	0	1
A015	Afro	*BRCA2*	2929delC	c.2701delC	p.Ala902Leufs	0	0	1^j^	2^m^	0	3	13	16
*Variants of uncertain significance*													
A032	Afro	*BRCA2*	—	c.9259C>A	p.Leu3087Ile	0	0	1^j^	0	0	1	2	3
A038	Raizal	*BRCA2*	1778A>G	c.1550A>G	p.Asn517Ser	0	2	1^j^	1^n^	0	4	16	20
A058	Afro	*BRCA2*	—	c.3685G>A	p.Val1229Ile	0	0	1^j^	0	0	1	2	3

The novel mutation is marked in bold.

BIC, Breast Cancer Information Core database as of November 2020 (https://research.nhgri.nih.gov/bic/).

Nomenclature follows Human Genome Variation Society (HGVS) (https://www.hgvs.org/).

African descent was used for the African-American population.

Hispanic descent was used for individuals of Spanish, Mexican, Central and South American, Cuban, or Puerto Rican descent.

Unknown, no information available.

Total reported in BIC and those from the present study.

ClinVar as of November 2020 (https://www.ncbi.nlm.nih.gov/clinvar/).

Total from BIC, ClinVar, and those from the present study.

Mutation previously reported.^15^

Mutations identified in the present study.

Novel mutation identified in the present study.

Reported in 2 patients with BC from Colombia.^28^

Reported in 2 White-mestizo families from Colombia^11^ and Spain^25^ with multiple breast cancer cases.

Reported in the Spanish population.

The intronic *BRCA1*_c.213-12A>G mutation was identified in an Afro-Colombian index patient diagnosed with BC at 37 years of age from a family featuring multiple BC cases from Bolivar (northern coast of Colombia). The proband’s mother was diagnosed with bilateral BC at the ages of 35 and 55 years and a deceased maternal aunt with BC at 30 years of age. Four other maternal family members were diagnosed with various cancer types: 2 with colon cancer at the ages of 41 and 60 years, and 2 with uterine cancer at the ages of 50 and 70 years (Supplementary Fig. S1A). The pathogenic *BRCA1*/c.4163_4164insA mutation was identified in an Afro-Colombian family affected by both breast and OC, with the proband diagnosed with BC at the age of 45, a deceased sister diagnosed with OC at the age 47, and a maternal female cousin diagnosed with BC at the age of 47. Three other family members were diagnosed with various cancer types: 2 with colon cancer at 71 years of age and another with uterine cancer at 58 years of age (Supplementary Fig. S1B). A novel pathogenic frameshift mutation, *BRCA1*_c.4203delT, was identified in an index patient diagnosed with BC at the age of 43 years from a family of the Raizal ethnic group from San Andres Island affected by multiple cases of BC. A deceased proband’s cousin on the maternal side was diagnosed with BC at the age of 45. Four other maternal family members were diagnosed with various cancer types: one with colon cancer at the age of 28 and 3 with uterine cancer at the ages of 25, 40, and 71 years (Supplementary Fig. S1C). The *BRCA2*_c.2701delC mutation was identified in an Afro-Colombian family affected by breast/OC, with the proband diagnosed with BC at the age of 40, a deceased aunt diagnosed with BC at the age of 50, and 2 maternal cousins diagnosed with BC at the age of 50 or OC at the age of 36 (already deceased; Supplementary Fig. S1D). The phenotypes of all families harboring deleterious *BRCA1/2* germline mutations are shown in Table 3.

**Table 3.** Characteristics of the Afro-Colombian families affected by breast/ovarian cancer harboring *BRCA1/2* mutations and variants.

Family	No. of cancers		Age at onset (years)		Other cancers: age at onset (years)
	Female BC (bilateral)	OC	Female BC	OC	
*Families carrying deleterious BRCA1 mutations*					
A004	3 (1)	—	37^a^, 35/55, 30	—	2× colon (41, 60), 2× uterus (50, 70)
A012	2	1	45^a^, 47	47	2× colon (71), uterus (58)
A035	3 (1)	1	30/33^a^, 52, 30	60	Brain (60), stomach (59), prostate (60)
A036	2	—	43^a^, 45	—	3× uterus (25, 40, 71), colon (28)
*Families carrying deleterious BRCA2 mutations*					
A015	3	1	40^a^, 50, 50	36	—
*Families carrying BRCA2 variants of uncertain clinical significance*					
A032	2	——	55^a^, 63	—	2× leukemia (75, 80), esophagus (70), colon (65), bone (50)
A038	2	—	56^∗^,?	—	Prostate (66)
A058	2	—	48^∗^, 53	—	Stomach (66), prostate (61), uterus (72)

Probands.

BC, breast cancer; OC, ovarian cancer.

Multiplex ligation-dependent probe amplification screening of the 55 index cases negative for small-scale *BRCA1/2* mutations did not reveal any LGRs.

**Figure 1.**
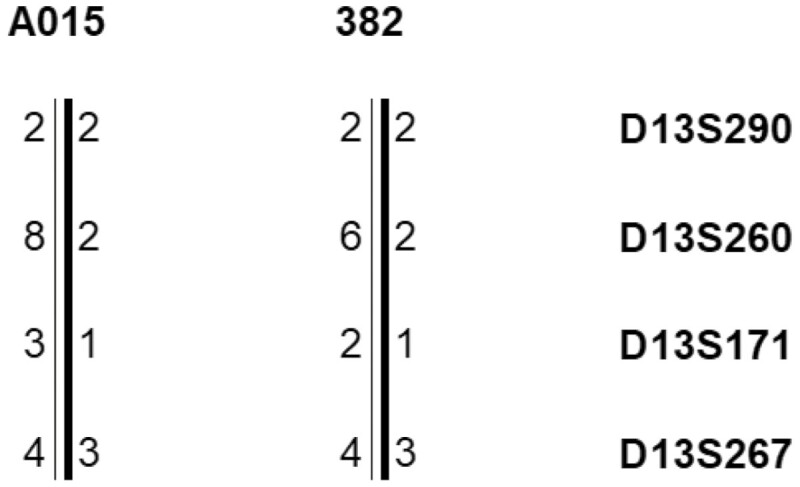
Haplotype analysis of breast cancer patients carrying the *BRCA2*_2929delC mutation at 4 microsatellite loci flanking the *BRCA2* gene. A015: Afro-Colombian carrier; 382: White/mestizo carrier.^11^ Alleles are coded by numbers. D13S290: allele 2 (CA)12, D13260: allele 2 (CA)19, allele 6 (CA)23, allele 8 (CA)25, D13S171: allele 1 (CA)13, allele 2 (CA)14, allele 3 (CA)15, D13S267: allele 3 (CA)34, allele 4 (CA)35. Common haplotypes are indicated by a bold bar.

Haplotype analysis of the recurrent *BRCA2*_c.2701delC mutation was performed on 2 mutation carriers (one Afro-Colombian BC patient identified in the present study and one previously identified White/mestizo BC patient ^11^) at 4 *BRCA2* flanking loci. The 2 *BRCA2*_c.2701delC carriers shared the same haplotype (Fig. 1).

## Discussion

This is the first report that describes the frequency and spectrum of small-scale mutations and LGRs in the *BRCA1/2* genes of patients with early-onset and familial breast and OC among Colombians of African descent. Four *BRCA1/2* pathogenic/likely pathogenic variants (including the recurrent *BRCA2*_c.2701delC mutation, which showed a founder origin) were identified, and one variation was already confirmed *(BRCA1*_c.5123C>A) in 60 Afro-Colombian families affected by breast and OC (8.3%). Overall, *BRCA1* mutations (80%) were more common than those in *BRCA2* (20%), which is similar to the findings observed in most Latin American countries.^19,20^ This is in line with a report describing that women of African (15.6%) and Latin American (14.8%) descent have a significantly higher prevalence of deleterious *BRCA1/2* mutations than women of Western European ancestry (12.1%), mainly due to an increased prevalence of *BRCA1* mutations in these 2 groups.^21^

It has been reported that African-American patients with BC are more likely to be affected at a younger age, have a more aggressive disease, and have a higher likelihood of dying from this disease than women from other ethnic groups.^22^ Additionally, it has been shown that African-American patients with BC harboring pathogenic *BRCA1/2* mutations are younger at the age of diagnosis than noncarriers (median ages: 37 years vs 47 years).^23^ This is consistent with what was observed in our Afro-Colombian study, where the median age of BC onset among *BRCA1/2* carriers was 40 and of 51 for noncarriers. In another study on the African-American population in Washington, DC, the median age of BC diagnosis was 38 in *BRCA2* carriers, whereas that of noncarriers was 47.^24^ This differs from the findings in the White/mestizo Colombian population, where there was no difference in the age of BC diagnosis between *BRCA1/2* carriers (median ages: 41 vs 45.5, respectively) and noncarriers (median age: 42).^12^ These results suggest that, to ensure diagnosis of BC at the earliest possible age among Afro-Colombian *BRCA1/2* mutation carriers, priority should be placed on giving access to genetic tests to this specific population group. This should be brought to the attention of health authorities because the healthcare of those of African descent in Latin America continues to be neglected compared with that of the general population.

The 2 *BRCA2*_c.2701delC mutation carriers (one Afro-Colombian BC patient from our study cohort and one White/mestizo BC patient previously reported^11^) shared a conserved haplotype at the 4 informative loci, implying that this mutation may have a common ancestor. This mutation was also previously identified in 2 families (one from Spain and one from Brazil).^25,26^ This finding does not allow us to infer the possible origin of this mutation because it was identified in both Hispanic and Afro-descendant populations. However, it is likely that this mutation arose during the colonization of Latin America.^27^

The intronic *BRCA1*_c.213-12A>G mutation identified in our cohort was previously described in 2 patients with BC from Antioquia (west of Colombia) at the ages of 30 and 39, respectively.^28^ Unfortunately, data on the ethnicity of the 2 mutation carriers were not provided by the authors. However, given that Antioquia is one of the regions of Colombia with the highest proportion of Afro-Colombians^4^, it is possible that these 2 carriers were of African descent. This recurrent mutation should be further characterized in future larger-scale studies.

In our study, a novel *BRCA1*_c.4203delT mutation in a BC patient from San Andres Island was identified, where the highest BC incidence and mortality rates occur (along with the Caribbean coast and Cali).^29,30^ These results could be explained by the diverse genetic admixture that characterizes the Raizal population. Furthermore, the high incidence and mortality rates of BC have been linked to precarious socioeconomic conditions, poor healthcare systems, limited access to medications, and shortages of care facilities and genetic counseling; collectively, these conditions are often common among less favored populations, which frequently correspond to islands and departments off the coast of Colombia.

## Conclusion

Of the 5 pathogenic Afro-Colombian mutations, 2 mutations, *BRCA1*_c.5123C>A ^12^ and *BRCA2*_c.2701delC, were previously reported in White/mestizo patients with BC,^11^ whereas the other 3 *BRCA1* mutations, *BRCA1*_c.213-12A>G, c.4163_4164insA, and c.4203delT, were only detected in Afro-Colombians. The spectrum of mutations of the *BRCA1/2* genes could thus vary by ethnic group. Despite the small sample size and the number of pathogenic mutations identified, our findings may point to differences in the *BRCA1/2* mutation spectrum between these 2 population groups from Colombia.

## Supplementary Material

oyab026_suppl_Supplementary_Figures

oyab026_suppl_Supplementary_Tables

## Data Availability

The data underlying this article will be shared on reasonable request to the corresponding author.
