# Supplementary material for: Prevalence of BRCA1 and BRCA2 Germline Mutations in Patients of African Descent with Early-Onset and Familial Colombian Breast Cancer
Source: Oncologist. 2022 Feb 15;27(2):e151–7. doi: 10.1093/oncolo/oyab026 (PMC8895486; doi:10.1093/oncolo/oyab026)
Supplement: oyab026_suppl_Supplementary_Figures [file oyab026_suppl_supplementary_figures.pdf]

The Oncologist
The official journal of the Society for Translational Oncology

Supplemental Figures for:
Prevalence of BRCA1 and BRCA2 Germline Mutations in Early-Onset and Familial Colombian Breast Cancer Patients of African Descent
Victoria E Villegas et al.

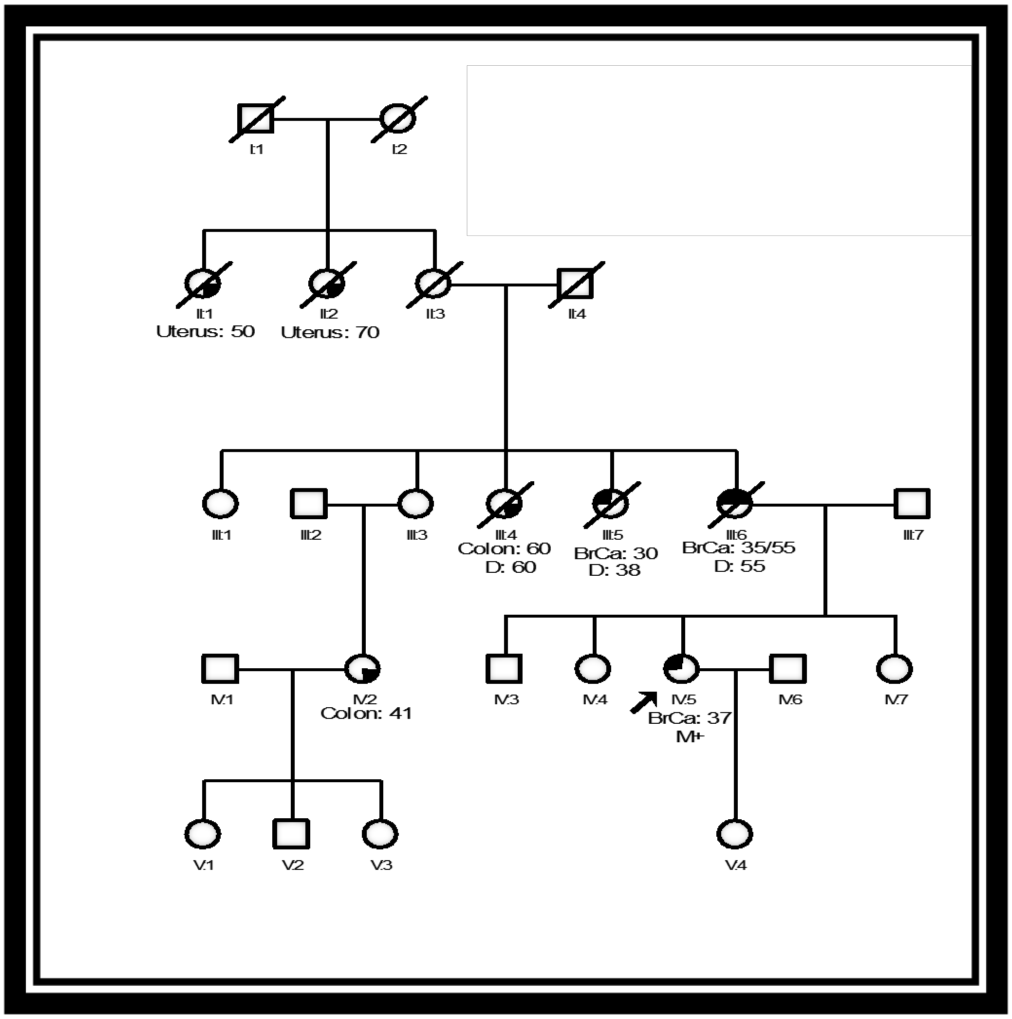


**Figure S1A:** Pedigree of the breast cancer-affected Family A004. The index patient is marked with an arrow. Circles are females, squares are males, and a diagonal slash indicates a deceased individual. Symbols with filled left upper quadrant: unilateral breast cancer. Symbols with filled right and left upper quadrants: bilateral breast cancer. Symbols with filled right lower quadrant: cancer other than breast cancer (the name of that cancer is mentioned). Identification

numbers of individuals are below the symbols. D, death; BrCa, breast cancer. The numbers following these abbreviations indicate age at cancer diagnosis. M+, mutation-positive for a pathogenic/likely pathogenic variant.

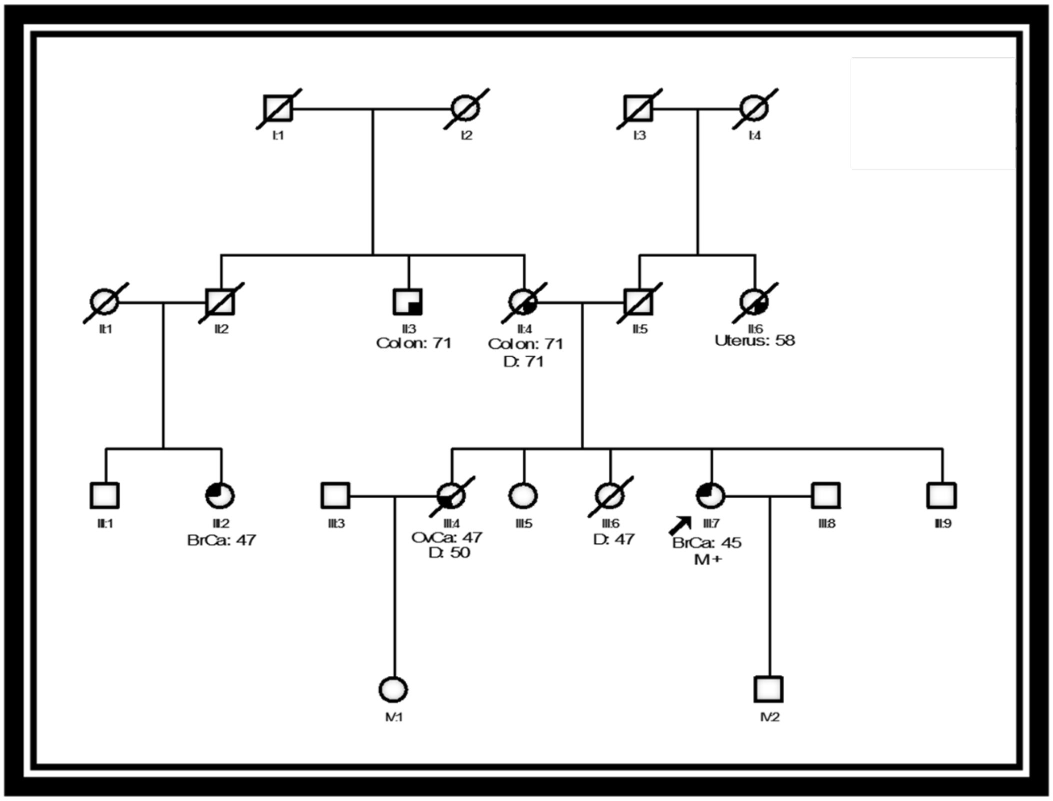


**Figure S1B:** Pedigree of the breast/ovarian cancer-affected Family A012. The index patient is marked with an arrow. Circles are females, squares are males, and a diagonal slash indicates a deceased individual. Symbols with filled left upper quadrant: unilateral breast cancer. Symbols with filled left lower quadrant: ovarian cancer. Symbols with filled right lower quadrant: cancer other than breast cancer (the name of that cancer is mentioned). Identification numbers of individuals are below the symbols. D, death; BrCa, breast cancer; OvCa, ovarian cancer. The numbers following these abbreviations indicate age at cancer diagnosis. M+, mutation-positive for pathogenic/likely pathogenic variant.

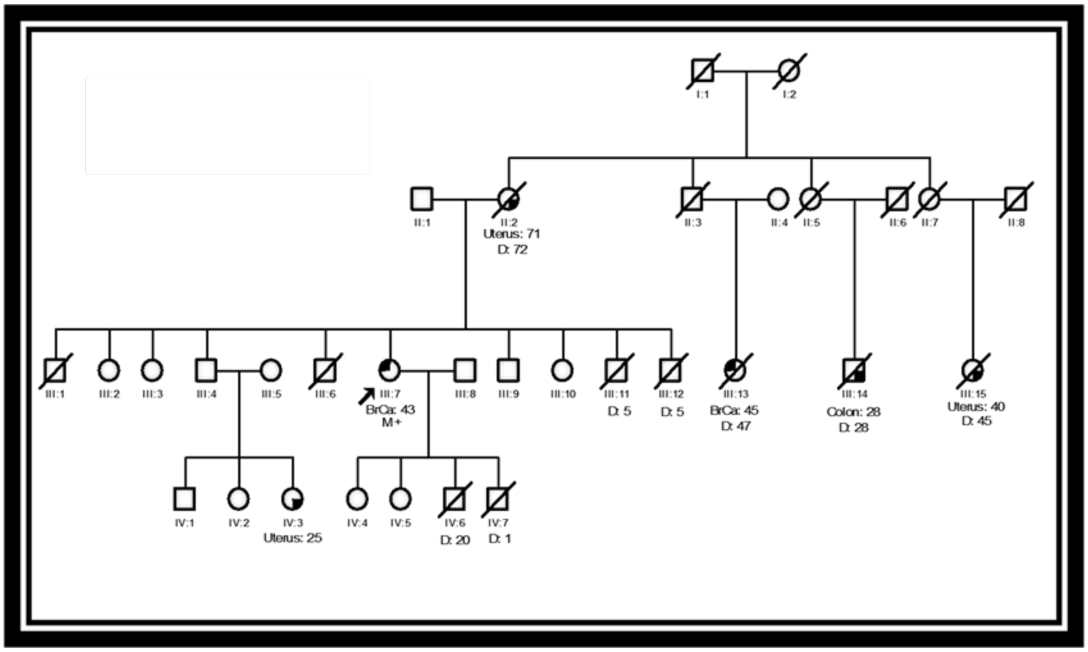


**Figure S1C:** Pedigree of the breast cancer-affected Family A036. The index patient is marked with an arrow. Circles are females, squares are males, and a diagonal slash indicates a deceased individual. Symbols with filled left upper quadrant: unilateral breast cancer. Symbols with filled right lower quadrant: cancer other than breast cancer (the name of that cancer is mentioned). Identification numbers of individuals are below the symbols. D, death; BrCa, breast cancer. The numbers following these abbreviations indicate age at cancer diagnosis. M+, mutation-positive for a pathogenic/likely pathogenic variant.

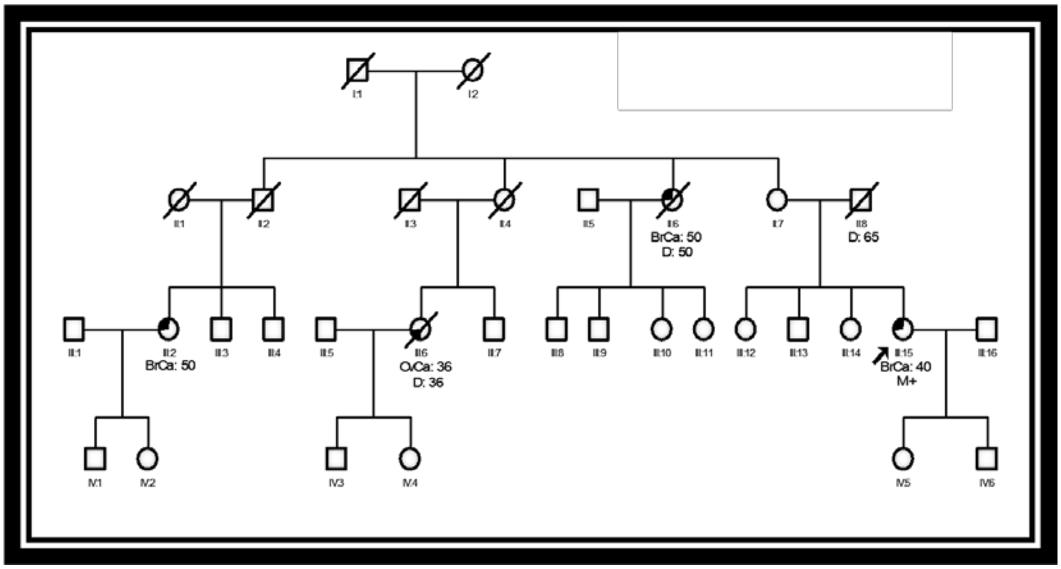


**Figure S1D:** Pedigree of the breast/ovarian cancer-affected Family A015. The index patient is marked with an arrow. Circles are females, squares are males, and a diagonal slash indicates a deceased individual. Symbols with filled left upper quadrant: unilateral breast cancer. Symbols with filled left lower quadrant: ovarian cancer. Identification numbers of individuals are below the symbols. D, death; BrCa, breast cancer; OvCa, ovarian cancer. The numbers following these abbreviations indicate age at cancer diagnosis. M+, mutation-positive for pathogenic/likely pathogenic variant.
