# Supplementary material for: Prevalence of BRCA1 and BRCA2 Germline Mutations in Patients of African Descent with Early-Onset and Familial Colombian Breast Cancer
Source: Oncologist. 2022 Feb 15;27(2):e151–7. doi: 10.1093/oncolo/oyab026 (PMC8895486; doi:10.1093/oncolo/oyab026)
Supplement: oyab026_suppl_Supplementary_Tables [file oyab026_suppl_supplementary_tables.pdf]

The Oncologist
The official journal of the Society for Translational Oncology

Supplemental Table for:
Prevalence of BRCA1 and BRCA2 Germline Mutations in Early-Onset and Familial Colombian Breast Cancer Patients of African Descent
Victoria E Villegas et al.

#CHROM[a]	POS[b]	ID	REF[c]	ALT[d]	QUAL	FILTER	INFORMATION	GT[k]:DP[l]	SAMPLE
**Deleterious *BRCA1/2* mutations**									
chr17	41256985	rs80358163	T	C	12785.8	PASS	AF=51.7[e];COV=1321[f];A=1[g];C=682[h];G=0[i];T=638[j];DEL=0	0/1:1320	004
chr17	41242982	rs80357788	C	CT	7534.73	PASS	AF=51.2;COV=783;A=0;C=783;G=0;T=0;DEL=0	0/1:782	012
chr13	32911192	rs397507637	TC	T	19803.7	PASS	AF=47.2;COV=1991;A=1;C=0;G=3;T=1987;DEL=0	0/1:1983	015
chr17	41215920	rs28897696	G	T	33732.8	PASS	AF=47.9;COV=3916;A=3;C=1;G=2040;T=1872;DEL=0	0/1:3912	035
chr17	41234574	.	GT	G	9803.0	PASS	AF=48.6;COV=1440;A=0;C=0;G=1440;T=0;DEL=0	0/1:1437	036
**Variants of uncertain significance**									
chr13	32968828	.	C	A	350.77	PASS	AF=33.3;COV=72;A=24;C=48;G=0;T=0;DEL=0	0/1:72	032
chr13	32907165	rs80358439	A	G	403.77	PASS	AF=51.1;COV=5075;A=2478;C=1;G=2594;T=2;DEL=0	0/1:5072	038
chr13	32912177	rs786203538	G	A	2368.77	PASS	AF=44.7;COV=295;A=132;C=0;G=163;T=0;DEL=0	0/1:295	058

Table S1. Summary data for the *BRCA1/2* mutations obtained from VCF files

Fileformat=VCFv4.2; [a]Crhomosome, [b]Position, [c]Reference allele, [d]Alternative allele.
[e]INFO=<ID=AF,Number=A,Type=Float,Description="Allele Frequency">
[f]INFO=<ID=COV,Number=A,Type=Integer,Description="Coverage">
[g]INFO=<ID=A,Number=A,Type=Integer,Description="Coverage on A">
[h]INFO=<ID=C,Number=A,Type=Integer,Description="Coverage on C">
[i]INFO=<ID=G,Number=A,Type=Integer,Description="Coverage on G">
[j]INFO=<ID=T,Number=A,Type=Integer,Description="Coverage on T">
[k]FORMAT=<ID=DP,Number=1,Type=Integer,Description="Read Depth">
[l]FORMAT=<ID=GT,Number=1,Type=String,Description="Genotype”
